# Tinea nigra caused by *Cladosporium halotolerans*: first molecularly confirmed locally acquired case in Chile

**DOI:** 10.1016/j.mmcr.2026.100824

**Published:** 2026-10-06

**Authors:** Cristián Cádiz-Bosch, Sebastián Casilla, Beatriz Ramos, Eduardo Álvarez, Daniel Velásquez, Khanty Loyola

**Affiliations:** a Faculty of Medicine, Universidad del Desarrollo, Clínica Alemana de Santiago, Santiago, Chile; b Laboratory of Mycology, Institute of Biomedical Sciences, Faculty of Medicine, Universidad de Chile, Santiago, Chile; c Department of Dermatology, Clínica Alemana de Santiago, Santiago, Chile; d Universidad Finis Terrae, Santiago, Chile

**Keywords:** *Cladosporium halotolerans*, Tinea nigra, Dematiaceous fungi, Dermoscopy, Molecular identification

## Abstract

Tinea nigra is an uncommon superficial fungal infection that may clinically mimic melanocytic lesions. We report a case of tinea nigra in a 61-year-old Chilean male presenting with asymptomatic pigmented acral macules on the toes. Dermoscopic examination showed a reticulated pigmentation pattern with a pseudo-parallel ridge pattern. Direct microscopic examination revealed pigmented septate hyphae, and fungal culture was positive. Molecular identification by sequencing of the actin and internal transcribed spacer region confirmed *Cladosporium halotolerans* as the etiological agent. To our knowledge, this represents the first molecularly confirmed case of tinea nigra caused by *C. halotolerans* locally acquired in Chile. 2012 Elsevier Ltd. All rights reserved.

## 1 Introduction

Tinea nigra is an uncommon superficial fungal infection caused by melanized fungi, typically presenting as an asymptomatic brown to black macule on acral skin, which may clinically mimic melanocytic lesions [1]. The disease has been classically attributed to *Hortaea werneckii*; however, other dematiaceous fungi have occasionally been reported as etiological agents [2]. Species of the genus *Cladosporium* are ubiquitous environmental fungi and are increasingly recognized as potential causes of human infection, although their pathogenic role remains underreported [3]. Accurate species-level identification of *Cladosporium* is challenging because of marked morphological similarity and taxonomic complexity, making molecular methods essential for reliable identification [4]. Here, we report a locally acquired case of tinea nigra caused by *Cladosporium halotolerans* in a Chilean patient, confirmed by phylogenetic analysis, representing the first molecularly confirmed case in Chile.

## 2 Case

A 61-year-old Chilean man with an unremarkable medical history presented to the dermatology clinic on day 0 for evaluation of asymptomatic pigmented lesions on the feet. The lesions had been present for four months prior to consultation. He denied pain, pruritus, bleeding, history of trauma, recent travel, or systemic symptoms.

On day 0, physical examination revealed irregularly bordered, dark brown to black macules located on the lateral aspect of the third toe of the right foot and on the medial aspect of the fourth toe of the right foot (Fig. 1A and B). The lesions were flat, mildly scaled, and asymptomatic. Given their acral localization and pigmentation, a melanocytic lesion was considered in the differential diagnosis.

**Fig. 1:**
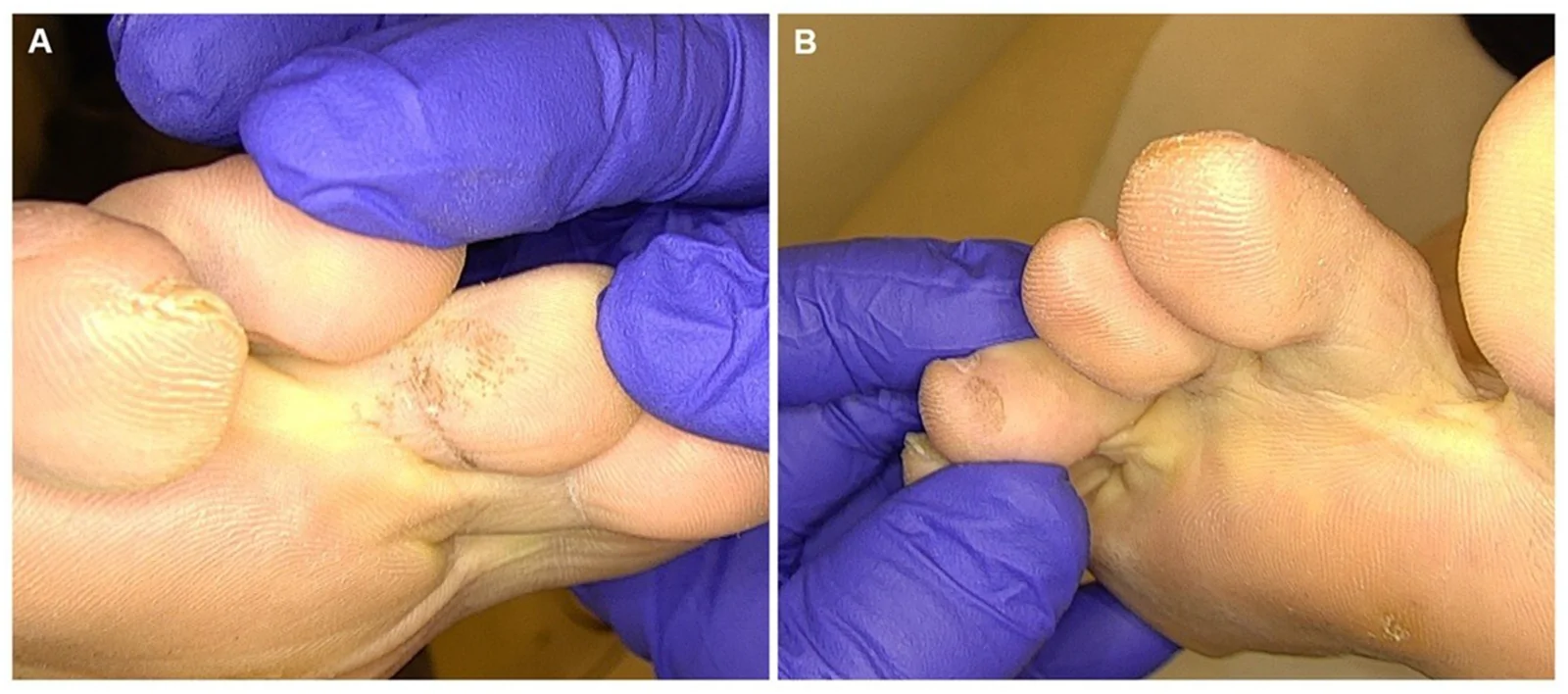
Clinical presentation. (A) Dark brown to black macule on the lateral aspect of the third toe of the right foot. (B) Similar lesion on the medial aspect of the fourth toe of the right foot.

Digital dermoscopic examination showed a reticulated and spiculated pigmented pattern, with focal alignment along the dermatoglyphics, without involvement of the acrosyringia (Fig. 2A and B). Based on these findings, a superficial fungal infection was suspected, and mycological studies were requested.

**Fig. 2:**
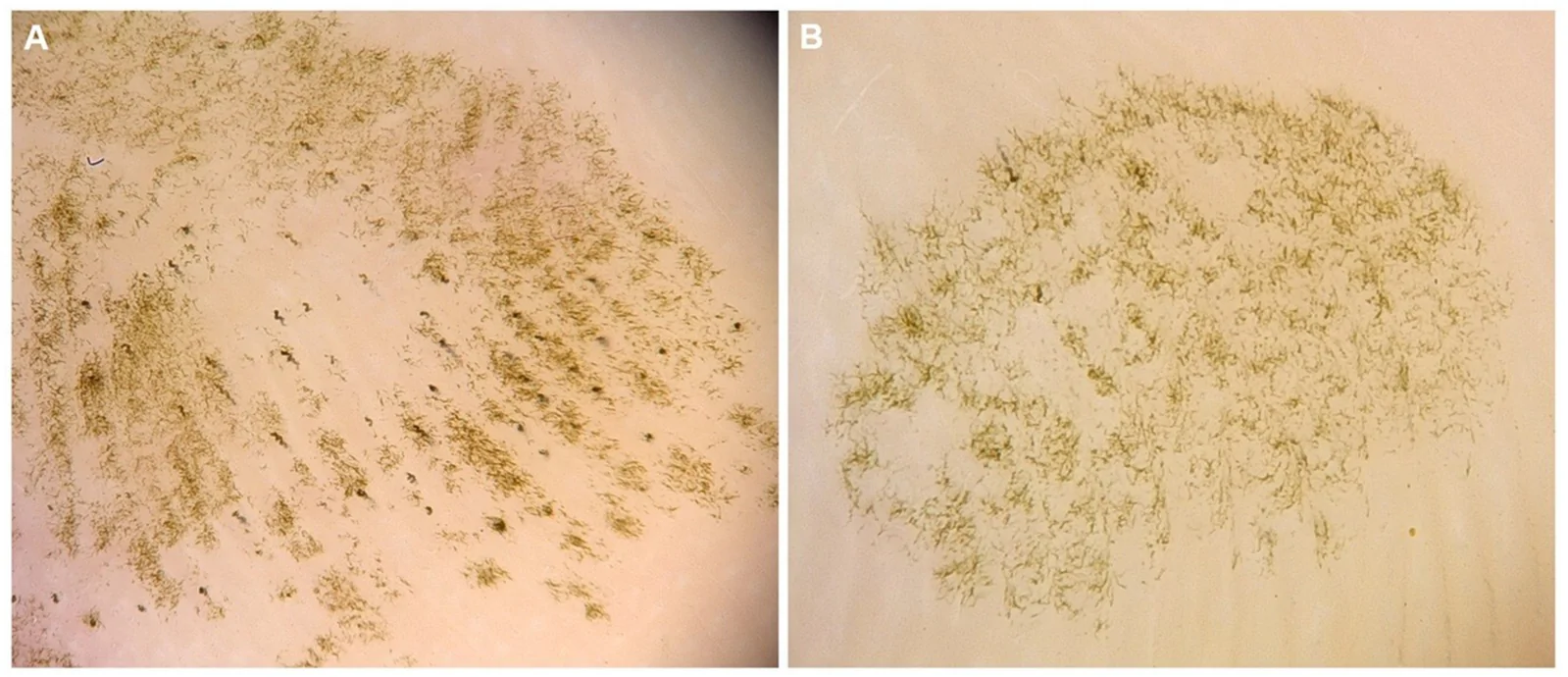
Dermoscopic findings. (A) Dermoscopy of the lesion on the third toe showing a reticulated and spiculated pigmentation pattern with focal alignment along the dermatoglyphics and sparing of the acrosyringia. (B) Similar dermoscopic findings in the lesion of the fourth toe.

Skin scrapings were obtained on day 0. Direct microscopic examination using a 10% potassium hydroxide preparation demonstrated abundant pigmented septate hyphae with an olive-green coloration, consistent with a dematiaceous fungus. Fungal cultures were performed on Sabouraud dextrose agar with chloramphenicol, Sabouraud dextrose agar with chloramphenicol and cycloheximide, and potato dextrose agar, and incubated at 25–30 °C for up to 4 weeks (Fig. 3A–C).

**Fig. 3:**
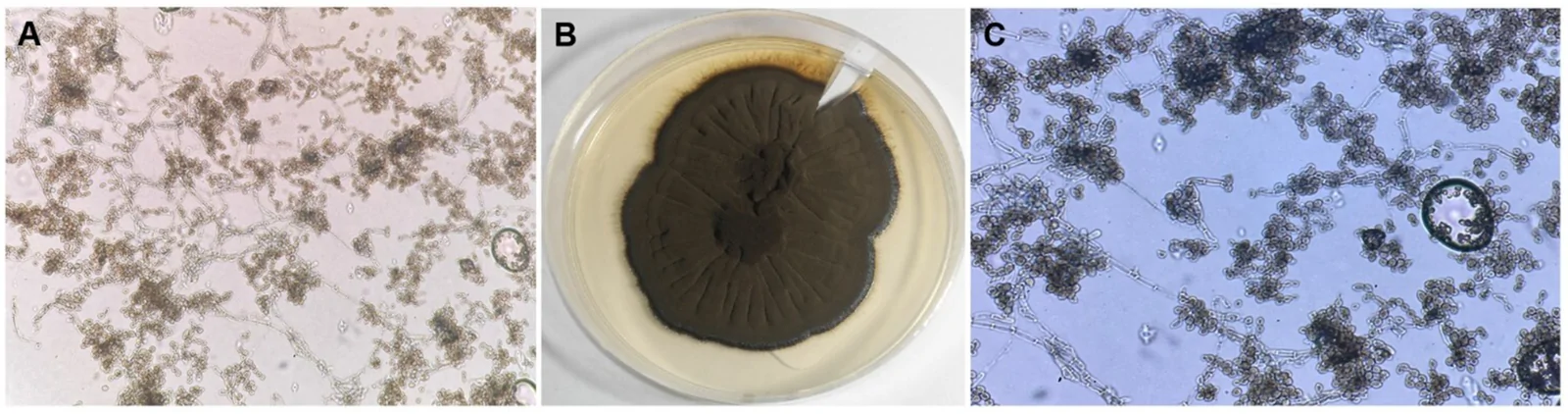
Mycological findings. (A) Direct microscopic examination (10% KOH, ×20) showing abundant pigmented septate hyphae with olive-brown pigmentation. (B) Fungal culture on Sabouraud dextrose agar with chloramphenicol showing velvety colonies with dark brown to olive-black pigmentation and radial zonation. (C) Microscopic examination of the culture (×40) showing pigmented septate hyphae and branched conidiophores producing chains of ellipsoidal to limoniform conidia, consistent with the genus *Cladosporium.*

For definitive identification, molecular analysis was performed. Fungal DNA was extracted, and the actin and internal transcribed spacer (ITS) region of ribosomal DNA were amplified and sequenced. The obtained sequences were compared with reference sequences available in GenBank. The ITS sequence showed 99.83% identity with *Cladosporium halotolerans* accession number MF473100.1, while the actin sequence showed 100% identity with *C. halotolerans* accession number OR068138.1, confirming the identification as *C. halotolerans* (Fig. 4). The sequences generated in this study were deposited in the GenBank database under accession numbers PZ923929 (ITS) and PZ980005 (actin).

**Fig. 4:**
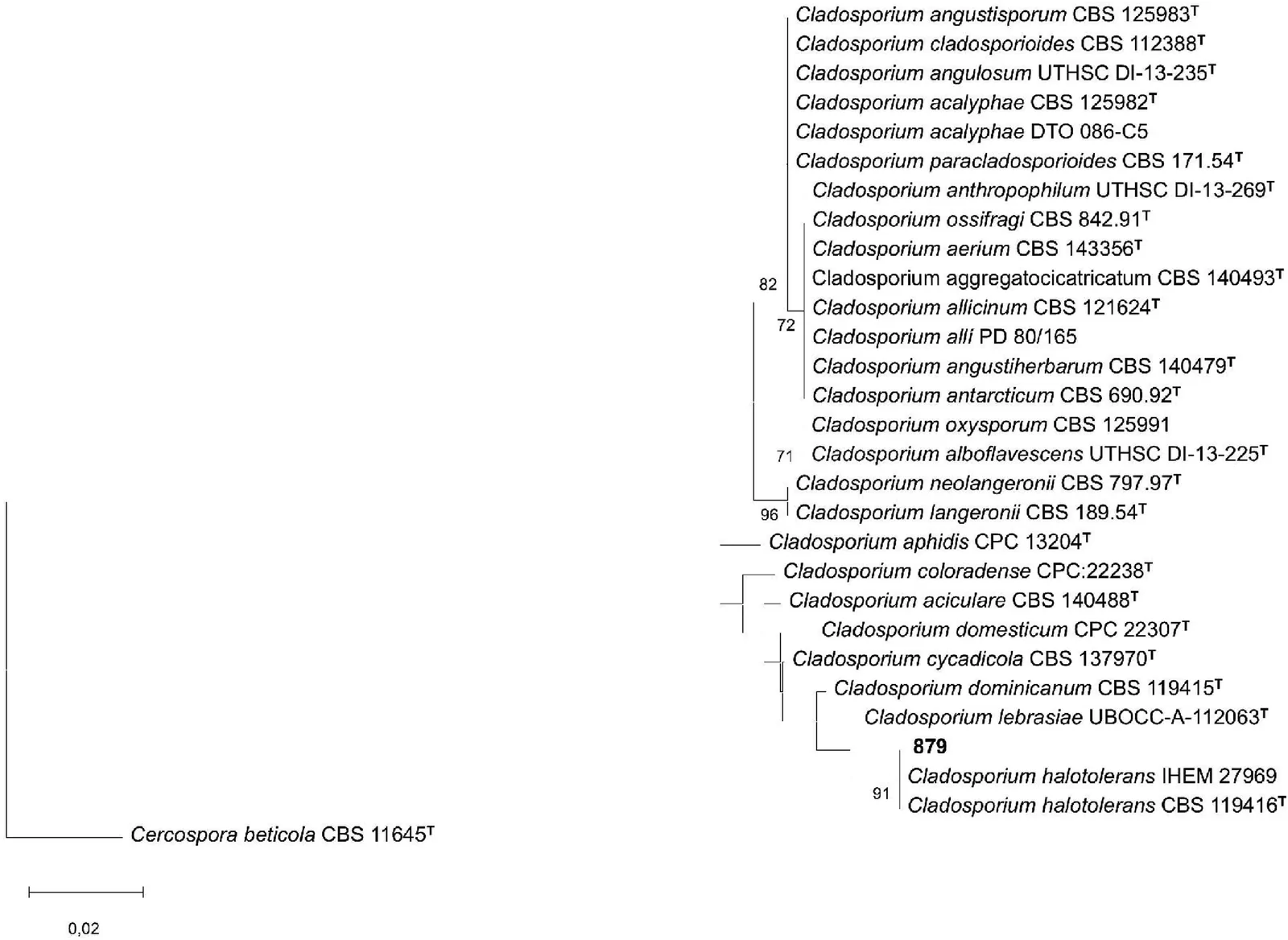
Phylogenetic analysis. Maximum likelihood phylogenetic tree based on ITS rDNA and actin gene sequences. Bootstrap support values (>70%; 1000 replicates) are shown at the nodes. *Cercospora beticola* CBS 11645^T^ was used as the outgroup. Strain 879 corresponds to the Chilean isolate from the present study.

Based on the clinical, dermoscopic, mycological, and molecular findings, a diagnosis of tinea nigra caused by *Cladosporium halotolerans* was established.

The patient was treated with a compounded topical preparation containing 3% salicylic acid and 10% urea, applied twice daily, in combination with topical terbinafine 1% every 12 hours for 4 weeks, achieving complete clinical resolution.

## 3 Discussion

Species of the genus *Cladosporium* have traditionally been considered environmental contaminants; however, several studies have demonstrated their ability to cause human disease, including superficial cutaneous infections [5,6]. Among them, *Cladosporium halotolerans* has been identified as one of the species most frequently recovered from clinical samples, supporting its pathogenic potential and suggesting that infections caused by this fungus may be underrecognized [6,7]. Although reports of invasive infections caused by *Cladosporium* species remain exceedingly rare, *C. halotolerans* has been implicated in a limited number of human infections, including chromomycosis, deep phaeohyphomycosis, and onychomycosis, further supporting its role as a human pathogen [6,[8], [9], [10]].

In this context, tinea nigra caused by *Cladosporium halotolerans* may represent a clinically underrecognized presentation of superficial pigmented acral lesions. Its clinical resemblance to melanocytic conditions underscores the importance of dermoscopy in the diagnostic approach. Classically, tinea nigra shows a reticulated or spiculated pigmentation pattern on dermoscopic examination; however, the presence of a parallel ridge pattern has also been reported and may closely mimic acral lentiginous melanoma [11,12]. Awareness of these dermoscopic features, together with prompt mycological examination, may help avoid unnecessary invasive procedures and facilitate early diagnosis [13,14].

From an epidemiological perspective, the molecular identification of *C. halotolerans* in this case is noteworthy. Tinea nigra has been classically attributed to *Hortaea werneckii*, a halophilic fungus associated with hypersaline environments in tropical and subtropical climates [15]. In contrast, *C. halotolerans* has been described as a ubiquitous, halotolerant, and xerotolerant species, frequently isolated from indoor environments across different geographic regions [16]. Moreover, the genus *Cladosporium* is cosmopolitan and widely distributed in nature [4]. These ecological features may help explain the occurrence of an autochthonous infection in a temperate region such as central Chile.

Direct microscopic examination with potassium hydroxide remains a rapid and reliable diagnostic tool for infections caused by melanized fungi, typically revealing pigmented septate hyphae, whereas fungal culture allows isolation of the etiological agent, though interpretation may be challenging due to the environmental nature of *Cladosporium* species [5,6]. These limitations make definitive species-level identification difficult when relying solely on phenotypic methods. In this context, sequencing of the internal transcribed spacer (ITS) region is currently considered an essential tool for the reliable identification of *Cladosporium halotolerans* in clinical samples. This may partly explain the underrecognition of this species in earlier reports [7].

Therapeutic approaches reported for infections caused by *C. halotolerans* are heterogeneous and based on isolated cases, reflecting the absence of standardized treatment recommendations for this uncommon pathogen. Available data suggest that triazoles tend to show greater in vitro activity than polyenes or echinocandins [7]. Nevertheless, clinical response may be variable in deep infections, as illustrated by a refractory case treated with adjunctive aminolevulinic acid photodynamic therapy [9]. For superficial presentations such as tinea nigra, topical antifungal and keratolytic agents are usually sufficient, with high rates of clinical resolution.

In conclusion, this case highlights *Cladosporium halotolerans* as a rare but clinically relevant etiological agent of tinea nigra and underscores the importance of considering uncommon melanized fungi in the differential diagnosis of pigmented acral lesions. The combined use of dermoscopy, direct mycological examination, and molecular identification was essential to achieve an accurate diagnosis and to avoid misdiagnosis as a melanocytic lesion. Increased awareness of this entity may contribute to earlier recognition and appropriate management of similar cases.

## CRediT authorship contribution statement

**Cristián Cádiz-Bosch:** Conceptualization, Data curation, Formal analysis, Investigation, Methodology, Project administration, Visualization, Writing – original draft, Writing – review & editing, Data curation, Investigation, Writing – review & editing. **Sebastián Casilla:** Data curation, Investigation, Visualization, Writing – original draft, Writing – review & editing. **Beatriz Ramos:** Investigation, Writing – original draft, Writing – review & editing. **Eduardo Álvarez:** Data curation, Investigation, Methodology, Resources, Supervision, Validation, Writing – original draft, Writing – review & editing. **Daniel Velásquez:** Data curation, Investigation, Resources, Supervision, Writing – original draft, Writing – review & editing. **Khanty Loyola:** Conceptualization, Data curation, Investigation, Project administration, Resources, Supervision, Validation, Writing – original draft, Writing – review & editing.

## Conflict of interest

There are none.
